# On-Site Electrochemical Detection of Sulfentrazone Using a Fully 3D-Printed Portable Sensing Platform

**DOI:** 10.1021/acsomega.6c05449

**Published:** 2026-09-15

**Authors:** Jacqueline Marques Petroni, Jonas Raul Balbinoti, Elias Jorge, Osvaldo Cavalcante da Costa Neto, Rodrigo Amorim Bezerra da Silva, Magno Aparecido Gonçalves Trindade, Bruno Gabriel Lucca

**Affiliations:** † Institute of Chemistry, 54534 Federal University of Mato Grosso do Sul, Campo Grande, Mato Grosso do Sul 79074-460, Brazil; ‡ Occupational Health and Safety Management Systems, Industry Social Service Innovation Center, Campo Grande, Mato Grosso do Sul 79002-072, Brazil; § Occupational Health Department, Eldorado Brasil, Três Lagoas, Mato Grosso do Sul 79641-300, Brazil; ∥ Institute of Chemistry, 28119 Federal University of Uberlandia, Uberlandia, Minas Gerais 38400-902, Brazil; ⊥ Faculty of Exact Sciences and Technology, Federal University of Grande Dourados, Dourados, Mato Grosso do Sul 79804-970, Brazil; # Department of Fundamental Chemistry, Institute of Chemistry, Universidade de São Paulo, São Paulo, São Paulo 05508-900, Brazil

## Abstract

The widespread use of pesticides in agricultural activities has increased concerns regarding environmental contamination and occupational exposure, particularly through dermal contact. In this work, we report a portable and low-cost electrochemical sensing platform for on-site monitoring of sulfentrazone (SFT), a herbicide extensively used in eucalyptus cultivation. The proposed device consists of a fully 3D-printed miniaturized electrochemical system fabricated by multi-material printing using conductive and non-conductive filaments, enabling accessible and decentralized analysis. SFT detection was performed by square-wave voltammetry (SWV), exhibiting a single irreversible oxidation peak at +0.86 V (pH 6.5). Under optimized conditions, the platform showed a linear response from 1.1 to 101.1 μmol L^–1^, with a limit of detection of 0.50 μmol L^–1^ (obtained through IUPAC recommendations). The proposed approach demonstrated satisfactory accuracy and precision in spiked tap water samples, with recoveries ranging from 100 to 103% and RSD values ≤ 2.9% (n = 3). Additionally, the platform was successfully applied to porcine skin samples as a model for detecting dermal contamination, yielding recoveries between 81 and 111%. The combination of additive manufacturing and electrochemical sensing provides a simple, reliable, and field-deployable strategy for SFT monitoring in environmental and biological matrices, highlighting the potential of fully 3D-printed analytical platforms for occupational and environmental applications.

## Graphical abstract

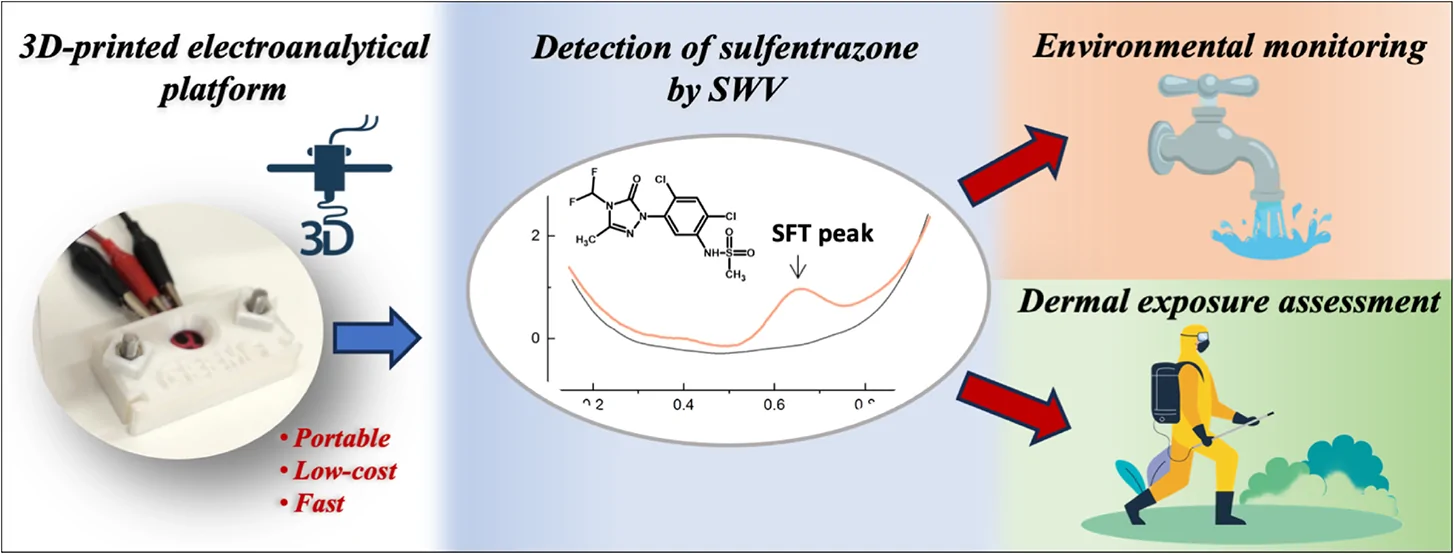

## 1 Introduction

Pesticides have been widely used for decades to protect crops against pests, diseases, and weeds, with herbicides representing the most extensively applied class worldwide.^1,2^ Sulfentrazone (SFT, Figure S1, Supplementary data) is a selective herbicide commonly employed in eucalyptus cultivation that acts by inhibiting protoporphyrinogen oxidase (PROTOX), an enzyme involved in the chlorophyll biosynthetic pathway.^3–5^

SFT is a weak acid (pKa = 6.56) whose solubility increases between pH 6.0 and 7.5.^5,6^ Its environmental behavior is strongly influenced by soil pH and organic matter content, exhibiting high persistence (43–280 days) and mobility in soil.^7,8^ These characteristics confer high leaching potential, favoring the contamination of soil and water bodies and posing risks to animal and human health.^6,9,10^ Human exposure may occur indirectly through contaminated environmental compartments or directly during pesticide handling and application.^11,12^ Among the exposure routes, dermal absorption is considered one of the most significant, mainly due to splashing, spillage, and spray drift during agricultural practices.^11,13^

Despite advances in agricultural technologies, monitoring strategies for evaluating occupational exposure to pesticides remain limited, particularly in developing countries.^14,15^ In the case of SFT, studies addressing the effects of prolonged human exposure are still scarce, although toxicological studies have demonstrated acute dermal toxicity in animal models.^16,17^ In this context, the development of rapid, low-cost, and on-site analytical methodologies is essential for improving environmental and occupational monitoring, especially considering the limitations associated with conventional laboratory-based analyses.

Chromatographic techniques remain the reference methods for pesticide analysis due to their high sensitivity and analytical performance. However, their application is often limited by the need for centralized laboratory infrastructure, expensive instrumentation, and time-consuming procedures.^18,19^ In this context, the growing demand for rapid and on-site analyses has driven the development of portable and cost-effective analytical alternatives capable of providing real-time results.^20–22^

Among these alternatives, electroanalytical methods have attracted considerable attention due to the portability of modern potentiostats and the possibility of miniaturizing electrochemical cells and electrodes. In addition to enabling fast and low-cost analyses, these methods are aligned with Green Chemistry principles by reducing reagent consumption, waste generation, and environmental impact.^23,24^ Furthermore, the versatility of electrochemical sensors, particularly through the use of different conductive materials and surface modifications, allows improvements in sensitivity and selectivity, making electroanalysis highly attractive for pesticide monitoring applications.^25^

Despite the environmental and toxicological relevance of SFT, only three previous studies have reported its electrochemical determination to date. Lima et al. employed glassy carbon electrodes, both bare and modified with multiwalled carbon nanotubes (MWCNTs), for SFT quantification and electrochemical degradation studies.^4^ Catrinck et al. reported SFT determination in soil samples,^3^ while Silva et al. developed an activated screen-printed electrode for the analysis of SFT in soy milk and groundwater.^26^ Although these studies demonstrated the feasibility of electrochemical detection, they were mainly based on conventional centralized laboratory configurations and benchtop instrumentation, limiting their applicability for in situ and on-site analyses. In addition, none of these previous works focused on detecting possible dermal contamination by SFT. Therefore, the development of portable, fully integrated, and low-cost electrochemical platforms for direct analysis in complex matrices remains an important challenge.

In this work, we address this limitation by developing a fully 3D-printed miniaturized electrochemical platform for the voltammetric determination of SFT in environmental and dermal samples. Additive manufacturing has emerged as a promising strategy for producing portable and integrated electrochemical devices with reduced fabrication costs, high reproducibility, and adaptable designs.^27–29^ The proposed platform employs conductive carbon black/polylactic acid (CB/PLA) electrodes integrated into a fully 3D-printed sensing system, enabling rapid and decentralized analysis. Particular emphasis was given to the possibility of dermal contamination monitoring as a strategy for assessing occupational exposure in agricultural environments. Additionally, the versatility of the platform enables its application to different matrices, including environmental samples and commercial formulations, offering a practical and accessible alternative to conventional laboratory-based analytical methods through simplified workflows and reduced operational costs.

## 2 Materials and Methods

### 2.1 Chemicals, Materials and Solutions

Chemicals of analytical grade were used without purification processes, as follows: acetic acid (purity ≥ 99.5%) and boric acid (purity ≥ 99.8%) were purchased from Dinâmica (Diadema, SP, Brazil); sodium hydroxide (purity ≥ 98.0%) and orthophosphoric acid (85% purity) were obtained from Acros Organics (Geel, Belgium). SFT analytical standard (96.8% purity) was supplied by Dr. Ehrenstorfer (Augsburg, Germany). The buffer and analyte solutions were prepared monthly with purified water (resistivity ≥ 18.2 MΩ cm) supplied by a Permution RO0310 reverse osmosis water purifier system (Permution, Curitiba, PR, Brazil). For the fabrication of the 3D-printed electrochemical system, filaments with a diameter of 1.75 mm were utilized. Polylactic acid (PLA) from 3DFila (Belo Horizonte, MG, Brazil) was used for the insulating structures, while a conductive carbon black/polylactic acid composite (CB/PLA) from Protopasta (Vancouver, WA, USA) was employed for printing the working (4 mm diameter), counter, and pseudo-reference electrodes. Mechanical components (M5 bolts and wing nuts) were purchased from local stores.

For the dermal exposure experiments, a solution of a commercial formulation containing 50% (w/w) SFT (FMC, Brazil) was prepared by dissolving it in tap water, in accordance with the manufacturer’s instructions provided in the product leaflet for eucalyptus cultivation. For the interference study, commercial pesticide products containing relevant active ingredients were used: acetamiprid (AC) and bifenthrin (BI) from UPL (Brazil); ammonium polyphosphate (APP) from Agrichem (Brazil); chlorfenapyr (CFN) and pyraclostrobin (PYR) from BASF (Brazil); glyphosate (GLY) from Monsanto (Brazil); isoxaflutole (ISX) from Bayer CropScience (Brazil); indaziflam (IDZ) from EnVu (Brazil); paraffinic hydrocarbons (PHC) from Energis 8 (Brazil); sulfluramid (SFR) from UNIBRÁS (Brazil); and thiamethoxam (TMX) from Syngenta (Brazil). The solutions were prepared by dissolving the commercial formulations in purified water according to the manufacturers’ recommendations for eucalyptus cultivation. The compounds with low water solubility (CFN and PYR) were dissolved in purified water:ethanol mixtures (3:2 and 1:1, v/v, respectively). Subsequently, mixed solutions containing SFT standard and each potential interferent were prepared, maintaining a concentration ratio 100 times lower than the recommended application rates, as shown in Table S1 (Supplementary Data).

### 2.2 Instrumentation and Measurements

The electrochemical measurements of SFT were performed at room temperature using a PalmSens potentiostat/galvanostat model EmStat4S (PalmSens BV, Utrecht, Netherlands) controlled by PSTrace software (version 5.9) and coupled to the 3D-printed portable electrochemical system. A Sovol SV04 independent dual extruder (IDEX) 3D printer (Sovol Technology, Shenzhen, Guangdong, China) was employed to produce the 3D-printed platform, which consisted of an electrochemical cell and a sensor, as previously reported by Conrado et al.^27^ The electrochemical cell was made of two separate parts: a platform, where the sensor is positioned and a lid with sealing ring, to hold the solutions on the sensor by mechanical pressure, as shown in Figure 1. The maximum capacity of this electrochemical cell is ca. 1 mL.

**Figure 1:**
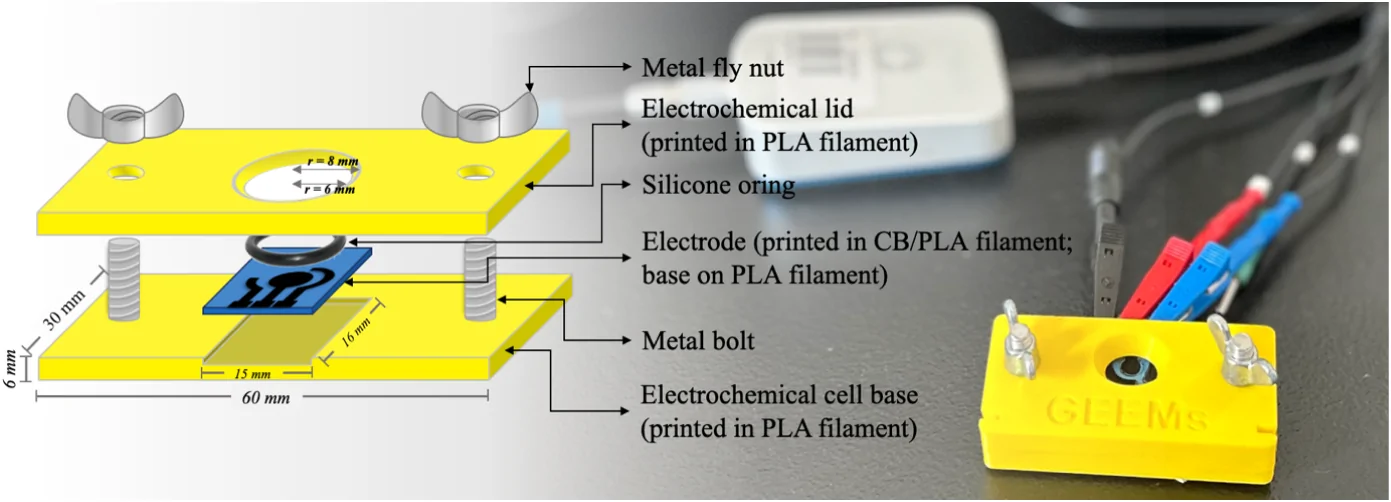
Scheme of disassembled components of 3D-printed electrochemical system and respective image after assembly.

### 2.3 Activation of the 3D-Printed Sensor

Prior to using the CB/PLA sensor, it underwent chemical and electrochemical activation processes based on previously reported methods.^30,31^ The chemical activation involved covering the surface of all three electrodes with a 1.0 mol L^–1^ NaOH solution for 30 min, followed by rinsing with purified water and drying with soft paper. Subsequently, the electrochemical activation of the working electrode was performed using amperometry, applying potentials of +1.4 V for 200 s and −1.0 V for 200 s (*vs.* CB/PLA) with the same NaOH solution. After activation, the system was rinsed with purified water and dried with soft paper.^27^

Additional information regarding the morphology and physicochemical properties of the activated CB/PLA electrode has been extensively reported in the literature owing to its widespread use. Comprehensive characterization, including scanning electron microscopy (SEM), energy-dispersive X-ray spectroscopy (EDS), thermogravimetric analysis (TGA), X-ray photoelectron spectroscopy (XPS), and electrochemical impedance spectroscopy (EIS), has been previously described by several authors.^27,29,32,33^ These studies provide a detailed understanding of the material and complement the content presented herein.

### 2.4 Quantitative Applications

#### 2.4.1 Addition Recovery and Simulated Dermal Exposure Studies

For the recovery assays, tap water samples collected in the city of Campo Grande, Mato Grosso do Sul, Brazil, were spiked with SFT standard at two concentration levels. The spiked samples were then diluted in 0.040 mol L^–1^ BR buffer solution (pH 6.5) in the ratio of 5:1 (v/v, buffer/sample) for electrochemical measurements.

A simulated quantitative experiment to detect possible dermal contamination was carried out using a powder-free disposable examination glove made of soft nitrile material, as shown in Figure 2A. Initially, a solution of commercial product (50% (w/w) SFT concentration) was prepared by dilution in tap water at the ratio of 1:200. Then, two swabs (plastic rod with rayon tip) were inserted inside the glove, which was subsequently inflated and its end tied. Next, the glove was immersed in the commercial product solution for subsequent collection.

**Figure 2:**
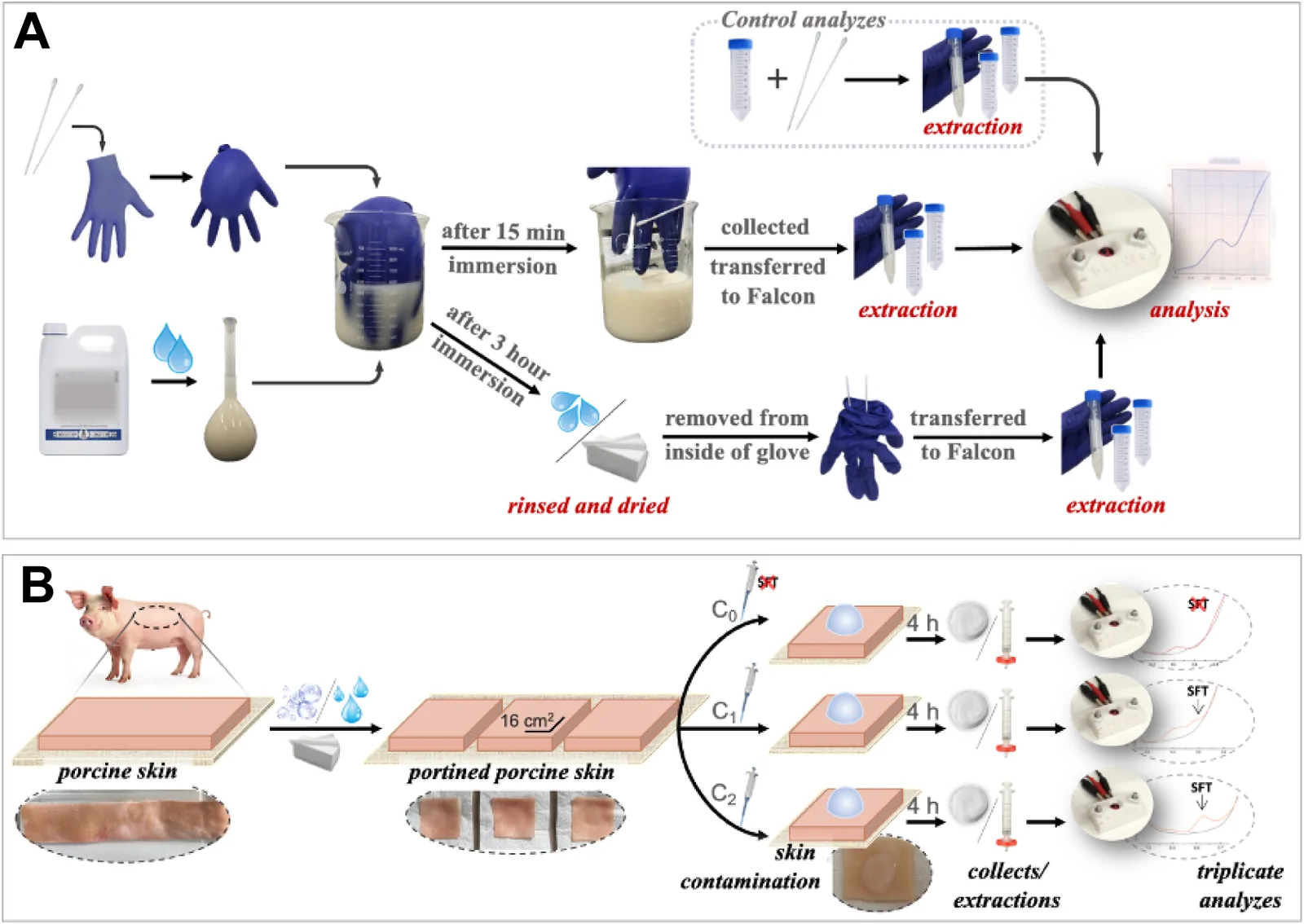
(A) Scheme of preparation and sample collection for quantification experiments to detect possible exposure. (B) Diagram for the spiking-collection steps for detection of SFT in exposed porcine skin.

First, collections were performed outside the glove 15 min after immersion using swabs. The swabs were transferred to Falcon tubes containing 10.00 mL of 0.040 mol L^–1^ BR buffer (pH 6.5) for extraction. An aliquot was then taken and added to the electrochemical cell for analysis. After 3 h, the glove was removed from the commercial product solution, rinsed with water, and dried with absorbent paper. The glove’s end was cut, and the swabs were removed from inside and placed in Falcon tubes with 2.00 mL of 0.040 mol L^–1^ BR buffer (pH 6.5) for extraction. An aliquot was then taken and added to the electrochemical cell for analysis. Control (blank) experiments were conducted using clean swabs, which were extracted with 2.00 mL of 0.040 mol L^–1^ BR buffer (pH 6.5) to ensure no interfering species were present from the swab itself. Measurements were performed in triplicate for both procedures.

#### 2.4.2 Application for Detection of SFT in Exposed Porcine Skin

To simulate occupational dermal exposure during agricultural practices, a skin exposure assay was performed. For this, porcine skin was utilized, as it is the most suitable model for human skin due to its histological similarity. The structural similarities include dermal collagen and elastin contents, as well as epidermal thickness and dermal-to-epidermal thickness ratios that are more similar to those of humans than those of other laboratory animals.^34,35^

The diagram in Figure 2B illustrates the steps followed for the SFT exposure experiments on porcine skin. Fresh dorsal porcine skin was purchased from a local commercial butcher shop as a food product. The skin was not obtained from animals specifically sacrificed for this research, and no experiments involving live animals were performed. The skin was cleaned with laboratory-grade neutral detergent solution and purified water, followed by disinfection with 70% ethanol and drying with a paper towel. The skin was then sectioned into three pieces measuring 4 cm × 4 cm and stored at room temperature under a paper towel. Aliquots of 500 μL and 1000 μL of SFT standard solutions, at concentrations of 50.1 μmol L^–1^ (C_1_) and 5.01 mmol L^–1^ (C_2_), respectively, were applied to two skin samples. A third piece received an aliquot of the solvent used for sample preparation (0.040 mol L^–1^ BR buffer, pH 6.5) to serve as a control (C_0_). Sample collection was performed 4 h after application using small cotton balls handled with tweezers. The cotton balls were transferred to syringes coupled to 0.22 μm filters, where 1 mL of electrolyte was added and the extracts were collected. Aliquots of each extract were then used for electrochemical analysis, with measurements conducted in triplicate.

## 3 Results and Discussion

### 3.1 Electrochemical Behavior of SFT

The electrochemical behavior of SFT on the surface of the 3D-printed sensor was initially investigated by cyclic voltammetry (CV) in 0.10 mol L^–1^ Britton-Robinson buffer at pH 5.5. As shown in Figure 3, the voltammogram exhibited a single oxidation peak at *E*_*p*_ = +0.64 V, with no cathodic peak observed in the reverse scan, indicating an irreversible electron transfer process. This behavior is consistent with previous reports for unmodified commercial carbon screen-printed electrodes.^3,4,26^ Furthermore, Lima et al. proposed a possible electrochemical oxidation pathway for SFT at a glassy carbon electrode. According to their study, the irreversible process involves two electrons and the oxidation of the methyl group attached to the heterocyclic ring, followed by the opening of the triazole ring.^4^ This proposed reaction mechanism is illustrated in Figure S2 (Supplementary Data).

**Figure 3:**
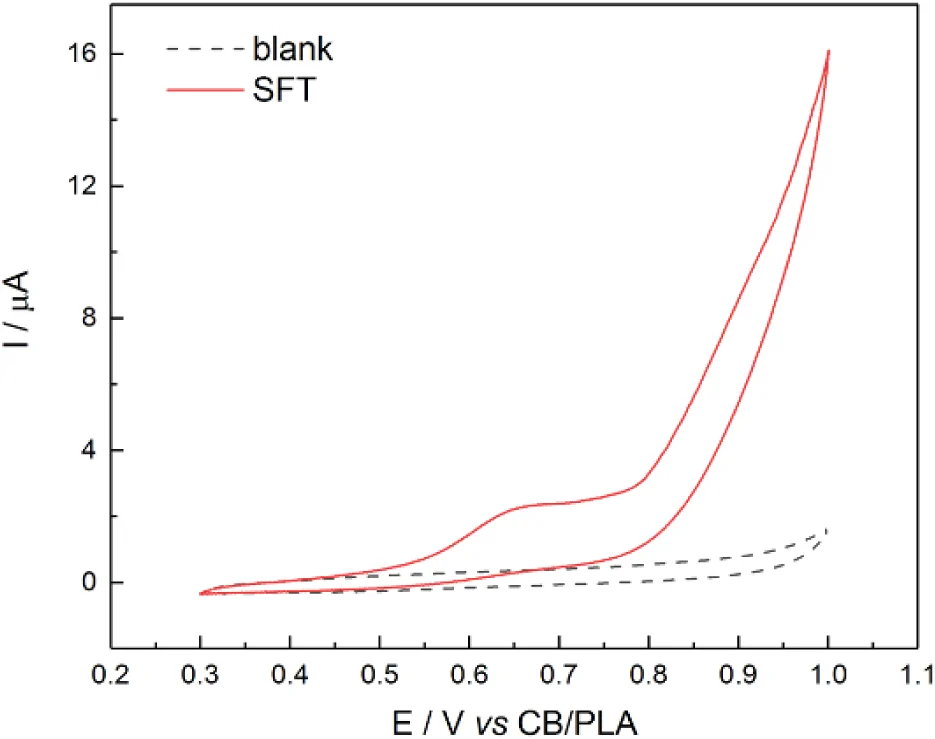
Cyclic voltammograms recorded in the absence (dashed line) and presence (solid line) of 1.0 mmol L^–1^ SFT in 0.10 mol L^–1^ BR buffer (pH 5.5). Scan rate was 100 mV s^–1^.

The influence of pH on the oxidation peak was evaluated over the range of 2.0 to 11.0 using 0.040 mol L^–1^ Britton-Robinson buffer solution (optimized concentration). As shown in Figure S3 (Supplementary Data), the peak potential (*E*_*p*_) exhibited negligible variation from pH 2.0 to 7.0 and a shift toward more positive values from pH 7.0 to 12.0. The peak current (*I*_*p*_) increased up to a maximum at pH 6.5 and decreased after this value. Considering that SFT is a weak acid (pKa = 6.56) and exhibits high aqueous solubility between pH 6.0 and 7.5, it is suggested that the enhanced current response at pH 6.5 results from the greater availability of the protonated form of the compound due to increased solubility.^36,37^ At lower pH values, reduced solubility likely impairs the electrochemical response. Therefore, pH 6.5 was selected as the optimal condition for subsequent experiments, as it provided the highest current intensity among the pH values tested.^7^

The mass transport of SFT species was also investigated by recording cyclic voltammograms in the scan rate range of 10 to 150 mV s^–1^ (Figure S4A, Supplementary Data). From Figure S4B (Supplementary Data), a linear relationship of anodic peak current (*I*_*p*_) and the square root of the scan rate (v^1/2^) was verified, with determination coefficient (*R* ^2^) of 0.992, indicating that the oxidation process is diffusion-controlled. Similarly, as shown in Figure S4C (Supplementary Data), a linear dependence (*R* ^2^ = 0.996) was observed in the plot of logarithm of peak current versus logarithm of the scan rate. The value of slope found was 0.59, which suggests a mixed mass transport controlled mainly by diffusion but also with adsorptive contribution on SFT oxidation process.^38^ Besides that, the peak current reduced significantly after the second sweep, which corroborates with previous information on adsorptive phenomenon. Thus, the first cycle was used for analytical purposes. Although this adsorptive contribution may contribute to electrode surface fouling during repeated measurements, the proposed sensor is intended for disposable single-use applications. Consequently, this phenomenon does not compromise the analytical performance or practical applicability of the device.

### 3.2 Optimization of Electroanalytical Conditions for SFT Analysis

To establish the most sensitive analytical method for SFT detection, different voltammetric techniques were comparatively evaluated. Linear sweep voltammetry (LSV), differential pulse voltammetry (DPV), and square-wave voltammetry (SWV) were investigated at the optimized pH of 6.5. From the voltammograms in Figure S5 (Supplementary Data), SWV yielded the most well-defined signal with the highest peak current intensity, being 1.37 times greater than LSV (2.52 μA *vs* 1.84 μA) and 2.23 times greater than DPV (2.52 μA *vs* 1.13 μA). Consequently, SWV was selected as the optimal technique for methodology development. Subsequently, the SWV parameters (amplitude, frequency, and step potential) were optimized through univariate experiments to maximize the analytical signal. Initially, the amplitude (*a*) was varied from 10 to 100 mV, while the other parameters were kept constant. As shown in Figure S6A (Supplementary Data), the highest peak current was achieved at 70 mV, without significant peak potential shift or increase in peak width. Frequency (*f*) was then evaluated in the range of 10 to 60 Hz (Figure S6B, Supplementary Data), with the best response obtained at 25 Hz. Finally, the step potential (ΔEs) was varied from 1 to 10 mV (Figure S6C, Supplementary Data), with 7 mV providing the best peak definition while maintaining an acceptable peak width. The combination of these optimized parameters resulted in an effective scan rate of 175 mV s^–1^ for the SWV measurements, ensuring fast data acquisition while maintaining the electrochemical sensitivity required for the 3D-printed sensing platform.

### 3.3 Analytical Figures and Comparison with Previous Literature

The optimized parameters, summarized in Table S2 (Supplementary Data), were used in the construction of an analytical curve, represented in Figure 4, which showed a linear dependence over the concentration range from 1.1 to 101.1 μmol L^–1^ (*R* ^2^ = 0.999). The sensitivity was 0.067 ± 0.001 μA L μmol^–1^. The limit of detection (*LOD*) was determined experimentally by recording SW voltammograms under increasing concentrations of SFT until obtaining an analytical signal with an intensity of ca. three times the background noise level.^39^ Therewith, the *LOD* of 1.05 μmol L^–1^ (0.40 mg L^–1^) was obtained. The limit of quantification (*LOQ*) was calculated using the relationship *LOQ* = 3.3 × *LOD,* where the value of 3.48 μmol L^–1^ (1.34 mg L^–1^) was found.

**Figure 4:**
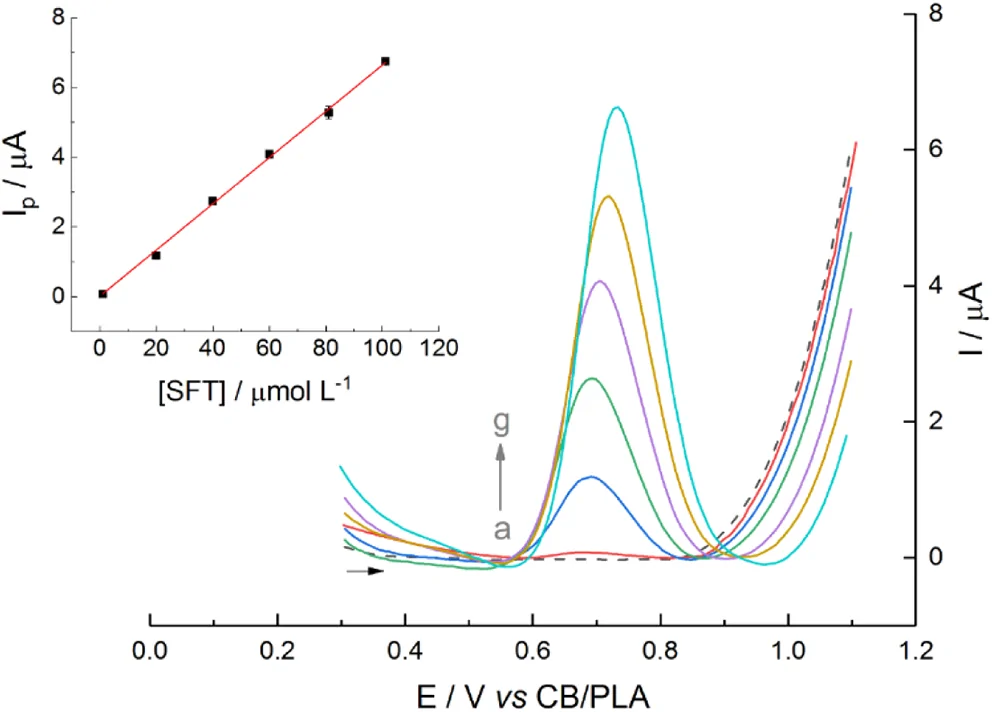
SW voltammograms in the absence (a, dashed line) and presence (b–g, solid lines) of 1.1 to 101.1 μmol L^–1^ of SFT. The inset presents the respective calibration plot (mean ± SD, n = 3). Experimental conditions as in Table S2.

The *LOD* and *LOQ* were also estimated according to the IUPAC recommendations using the calibration curve approach. The calculated *LOD*, obtained from the equation *LOD* = 3 × SD_*blank*_/*b*, was 0.50 μmol L^–1^ (0.19 mg L^–1^), while the *LOQ*, calculated as *LOQ* = 10 × SD_*blank*_/*b*, was 1.67 μmol L^–1^ (0.65 mg L^–1^), where SD_*blank*_ is the standard deviation of ten blank measurements and *b* is the slope of the calibration curve.^39^ The values of *LOD* and *LOQ* obtained here are among the lowest achieved in comparison with previously reported papers, as shown in Table 1.

**Table 1:** Comparison between This Work and Previous Reports Regarding Electrochemical Detection of SFT

Electrode	Technique	LOD (μM)	LOQ (μM)	Linear range (μM)	Miniaturized and portable apparatus	Integrated electrodes	Modification surface	Reference
**GCE** ^a^	DPV	17.2	66.0	1.0–230	No	No	No	^4^
**GCE-MWCNT** ^b^		2.6	8.8				Yes	
**GCE** ^a^	DPV	1.94	6.46	12.91–258.3	No	No	No	^3^
**CSPE-MWCNT** ^c^	SWV	0.15	0.51	1.00–30.0	No	Yes	Yes	^26^
CB/PLA^d^	SWV	0.5	1.67	1.1–101.1	Yes	Yes	No	This work

a Glassy carbon electrode.

b Glassy carbon electrode modified with multi-wall carbon nanotube.

c Carbon screen-printed electrode modified with multi-walled carbon nanotube.

d CB/PLA 3D-printed sensor; DPV: differential pulse voltammetry; SWV: square-wave voltammetry.

Notably, the sensor employed in this work features a miniaturized and integrated platform (comprising a three-electrode setup: working, counter, and pseudo-reference) that eliminates the need for additional materials (surface modifications) to achieve satisfactory electrochemical results, unlike most other studies. Furthermore, the 3D manufacturing method makes the sensor extremely versatile, allowing its design to be tailored to specific analysis needs and portability requirements. With a low manufacturing cost (ca. 15 US cents), the sensor is rapid (ca. 6 min) and easy to print, making it an attractive and easily implementable option for practical applications, especially in remote or resource-poor locations. Furthermore, the use of this 3D-printed sensor in conjunction with modern portable potentiostats provides a highly suitable portable analytical platform for on-site measurements, avoiding the need to send samples to centralized laboratories.

Another important point to highlight is that, according to the United States Environmental Protection Agency (EPA), exposure to SFT below 2000 mg kg^–1^ day^–1^ (equivalent to 5.16 mmol kg^–1^ day^–1^) is considered acceptable.^17^ Thus, the *LOD* achieved by the proposed sensor (0.50 μmol L^–1^) is significantly lower than this threshold, demonstrating the suitability of the methodology for detection and quantification of SFT in scenarios involving dermal exposure in agricultural workers.

### 3.4 Analytical Applications

#### 3.4.1 Recovery Assay in Water

The analytical performance of the proposed methodology was evaluated through the determination of SFT in two distinct water samples. As summarized in Table 2, SFT concentrations were absent or below the LOD in both samples. Thus, the samples were spiked with known concentrations of SFT and then submitted to the electrochemical analysis to evaluate the accuracy of the proposed method. The spiking values were based on acute dermal (*LD*_*50(dermal)*_ = 5.16 mmol kg^–1^ day^–1^) established by *EPA*.^17^ To ensure the sensor’s relevance for sub-toxic occupational monitoring, sample 1 was spiked at a level approximately 100-fold lower than the *LD*_*50(dermal)*_, while Sample 2 was spiked at half of that concentration. As shown in Table 2, the recovery values ranged between 100 and 103%, indicating the suitable accuracy for the 3D-printed sensing platform. Moreover, the relative standard deviations (*RSD*s) were ≤2.9%, indicating satisfactory precision and high reproducibility of the electrochemical response in complex aqueous matrices. These results demonstrate the suitability for the decentralized application of the method for determination of SFT in environmental samples, such as natural water matrices.

**Table 2:** Results Obtained from Recovery Assays in Samples of Tap Water and Porcine Skin

Samples		SFT spiked (μmol L^–1^)	SFT found^a^ (μmol L^–1^)	Recovery (%)	*RSD* (%)
Tap Water	1 and 2	0.0	<LOD	-	-
	1	58.1	59.9 ± 1.7	103.1	2.9
	2	29.0	29.3 ± 0.8	100.7	1.6
Porcine skin	C_0_	0.0	<LOD	–	–
	C_1_	50.1	55.7 ± 0.6	111.2	1.0
	C_2_	5000	4100 ± 8	81.0	0.2

a Average ± SD (n=3).

#### 3.4.2 Simulated Dermal Exposure: Addition-Recovery on Porcine Skin

Unintentional dermal exposure (whether accidental or occupational) to pesticides can occur in agricultural practices and can result in severe clinical symptoms. Strategies to monitor and control such exposure are essential to mitigate the physiological damage caused by herbicide contact. To simulate human skin contamination, an assay was performed using porcine skin as a biological model, following the protocol described in Section 2.4.2. SFT solutions at concentrations of 50.1 μmol L^–1^ (C_1_) and 5.01 mmol L^–1^ (C_2_) were applied to the porcine skin surfaces and samples were collected after a 4-hour interval to simulate a typical work shift. The samples were submitted to analysis using the standard addition method. From the voltammograms in Figure 5, no SFT oxidation peak was observed around +0.8 V for the control sample (C_0_), whereas distinct peaks were identified in the contaminated samples with SFT standard (C_1_ and C_2_). For the three samples, unidentified peaks near the SFT signal were observed. As the porcine skin was purchased commercially, the breeding, health status, and handling conditions of the animal prior to acquisition could not be controlled.

**Figure 5:**
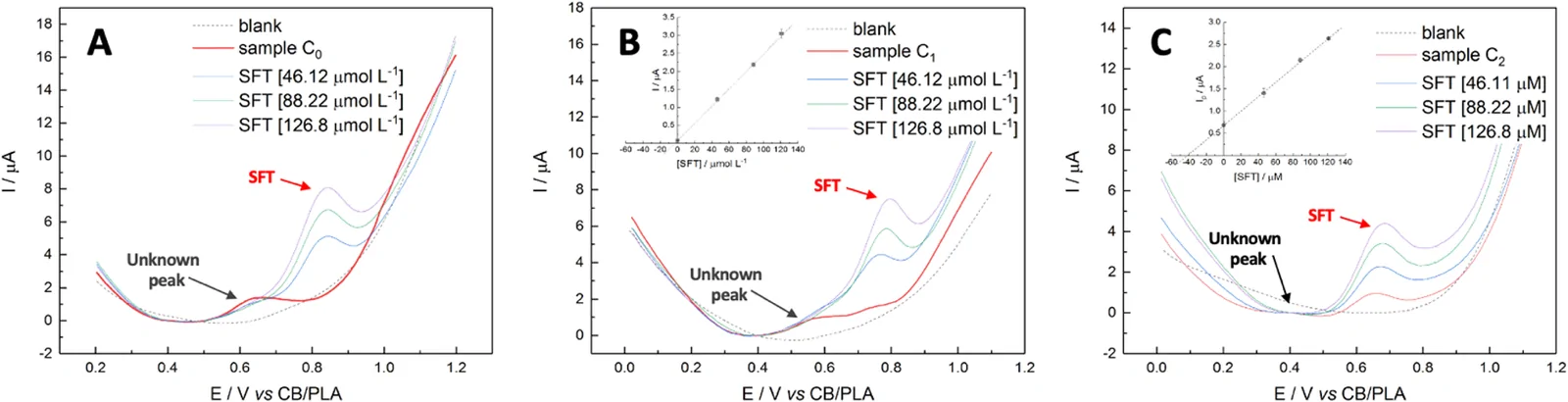
SW voltammograms obtained during the assay of dermal exposure for SFT quantification on porcine skin: A) control sample (C_0_), B) sample spiked with 50.10 μmol L^–1^ (C_1_) and C) sample spiked with 5.01 mmol L^–1^ (C_2_) of SFT standard. The inserted plots on (B) and (C) are the standard addition curves of the respective samples. Experimental conditions as in Table S2 (Supplementary Data).

The recovery values (obtained from standard addition curves) were between 81.0 and 111.2%, with *RSDs* ≤ 1.0%, as shown in Table 2. This recovery range is in accordance with the expected values accepted by the Clinical Laboratory Standards Institute (CLSI), which is between 80–120%.^40^ The low recovery value of the sample C_2_ can be attributed to a not so efficient collection or even losses caused in the sample extraction process since the *RSD* was extremely low (0.19%). Thus, the results obtained evidence a promising potential of the proposed method using CB/PLA electrodes for detection of dermal exposure to SFT. It is important to emphasize that the present study represents a proof of concept conducted under controlled experimental conditions. Therefore, further investigations involving specimens collected from individuals occupationally exposed to SFT will be necessary to fully establish the applicability of the proposed platform under real-world conditions.

#### 3.4.3 Application for SFT Detection in Simulated Contamination

To simulate possible contamination by handling herbicide during the workday using a nitrile glove, an assay was carried out as described in Section 2.4.1. The samples collected outside and inside the glove presented a characteristic peak of SFT close to +0.8 V, as shown in Figure 6A–B. The standard addition method was used to quantify SFT in these samples and the values obtained are summarized in Table 3. A control analysis was performed to ensure the reliability of results, where SFT was not detected (Figure 6C). The values found from the sampling outside of glove were compatible with the commercial dilution prepared, according to the information contained on the product label. For the result obtained for the sampling collected inside the glove, it is believed that the value found is due to the prolonged exposure time to the commercial product and the physical wear caused to the glove, by the distension of the material, which likely allowed a small amount of the herbicide solution to permeate through the glove material.

**Figure 6:**
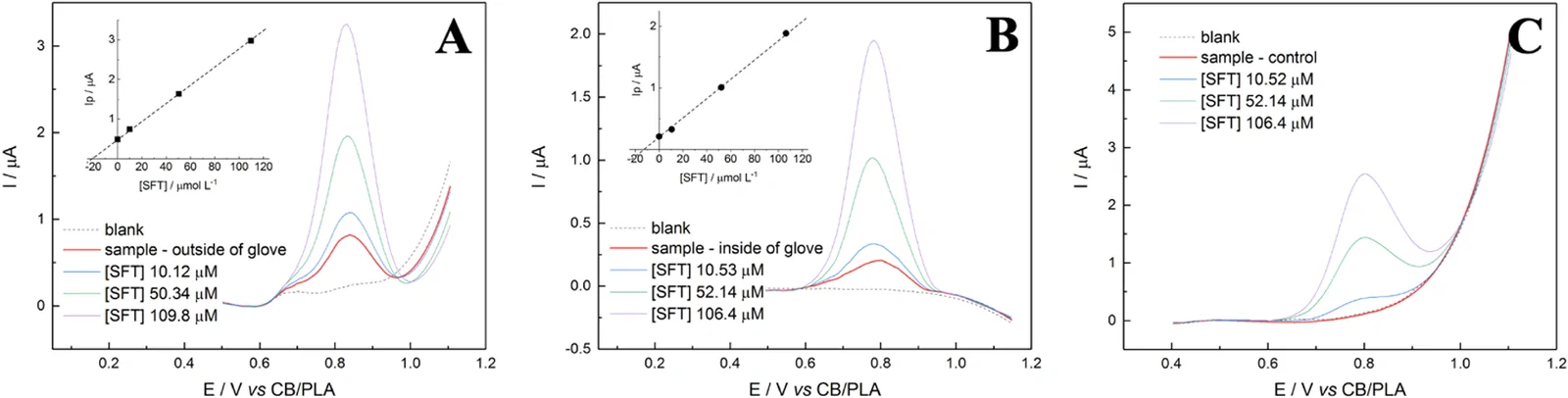
SW voltammograms obtained for the determination of SFT in commercial product solution collected outside (A) and inside (B) the glove and respective control experiment (C). Insets (A and B) are the respective standard addition curves. Experimental conditions as in Table S2 (Supplementary Data).

**Table 3:** Results of SFT Quantification in the Simulated Dermal Contamination Assay Using a Nitrile Glove

[SFT] in commercial solution (mmol L^–1^)	Sample collected	SFT found^a^ (mmol L^–1^)	*RSD* (%)
6.62	Outside of glove	6.5 ± 0.2	3.5
	Inside of glove	0.2 ± 9 × 10^–3^	3.7
–	Control	<LOD	–

a Average ± SD (n=3).

### 3.5 Effect of Possible Interferences

To simulate realistic potential interferences during on-site analyses of SFT, the selectivity of the method was assessed in the presence of commercial agrochemical formulations commonly applied alongside SFT in eucalyptus cultivation. The evaluated formulations contained active ingredients such as acetamiprid (ACT), bifenthrin (BIF), ammonium polyphosphate (APP), chlorfenapyr (CFN), pyraclostrobin (PYR), glyphosate (GLY), isoxaflutole (ISX), indaziflam (IDZ), paraffinic hydrocarbons (PHC), sulfluramid (SFR), and thiamethoxam (TMX). In addition to the active ingredients, commercial formulations may contain additives, adjuvants, diluents, and other components capable of altering the pH or solubility of the target analyte, thereby potentially affecting the analytical results.

To perform this selectivity assay, solutions of commercial agrochemical products were prepared according to the recommendations provided in their respective leaflets. SFT was then added to each solution at the concentration specified in its leaflet, simulating a possible mixture of products commonly used in the field. Table S1 (Supplementary Data) summarizes the pH values of each commercial product, the concentrations used in the experiments, and their ratios relative to SFT.

As shown in Figure S7A (Supplementary Data), the voltammograms revealed that none of the evaluated substances produced overlapping signals with SFT or exhibited electrochemical activity within the studied potential window. However, some variations in peak current and potential were observed in some samples, as illustrated in Figure S7B and S7C (Supplementary Data). The peak current responses for SFT in the presence of potential interferents ranged from 56.8% to 116.7% of the response obtained in the absence of interferents. Peak potential shifts ranged from 104.2% to 157.1% of the value obtained for the SFT control. These variations are likely attributed not only to the active ingredients but also to other formulation components such as additives, diluents, and adjuvants, which may alter solution pH or reduce analyte solubility. Overall, the results confirm that no qualitative interference with SFT detection was observed. However, commercial formulations containing acetamiprid, bifenthrin, and sulfluramid produced noticeable suppression of the oxidation current, indicating that matrix effects may influence quantitative analyses in complex mixtures. Therefore, for routine analyses of unknown field samples containing multiple agrochemicals, matrix-matched calibration or the standard addition method is recommended to compensate for possible matrix effects. The satisfactory recoveries obtained for representative agrochemical mixtures (Section 3.5.1) demonstrate that, despite these limitations, the proposed platform provides reliable quantitative results under some realistic application conditions. These findings underscore the potential of the SWV-based method for detection of SFT, even in more complex matrices containing multiple compounds.

#### 3.5.1 Effect of Potential Interferents in Quantitative Measurements

The effect of most commonly co-applied agrochemicals on the quantitative determination of SFT was also evaluated. For this purpose, two mixtures containing SFT and five agrochemicals frequently used in eucalyptus cultivation (GLY, APP, ISX, PHC and TMX) were prepared. The concentrations of each compound were selected to reflect the proportions typically employed under field conditions and are summarized in Table S3 (Supplementary Data).

The resulting mixtures, with identical agrochemical proportions but different SFT concentration levels, were subjected to recovery assays using the proposed sensing platform. To further demonstrate its portability and applicability, all measurements were performed on-site under actual field conditions, as illustrated in Figure S8A (Supplementary Data). Recovery values ranged from 97 to 103%, with *RSD* values ≤ 1.8% (Table 4), demonstrating the satisfactory accuracy of the proposed method even in the presence of commonly co-applied agrochemicals. These results further demonstrate that the proposed 3D-printed sensing platform is suitable for reliable SFT determination under realistic agricultural conditions, where multiple agrochemicals are commonly applied simultaneously. Representative voltammograms and the corresponding standard addition plot are shown in Figure S8B (Supplementary Data).

**Table 4:** Results Obtained for the Recovery Assays of SFT in the Presence of Other Common Agrochemicals

Sample	SFT added (μmol L^–1^)	SFT found (μmol L^–1^)^a^	Recovery (%)	*RSD* (%)
Mixture 1	10.7	11.1 ± 0.2	103.1	1.8
Mixture 2	21.5	20.9 ± 0.3	97.2	1.4

a Average ± SD (*n* = 3).

### 3.6 Stability, Reproducibility and Repeatability

The stability of SFT was assessed over a period of 10 consecutive days recording SWV measurements under optimized conditions. A single CB/PLA sensor was employed to perform daily measurements in a 53.8 μmol L^–1^ SFT solution, stored under refrigeration and analyzed at room temperature. As shown in Figure S9 (Supplementary Data), no significant variation was observed in the voltammetric response throughout the evaluated period. The relative standard deviation (*RSD*) for the peak currents was 6.3%, indicating good stability of both the analyte and the electrochemical system over time. Furthermore, these results provide additional evidence of the stability of the CB/PLA pseudo-reference electrode. Throughout the 10-day evaluation period, the oxidation peak potential exhibited negligible variation, with an *RSD* of 3.2%, indicating satisfactory potential stability.

To further assess the performance of the CB/PLA pseudo-reference electrode, its potential was compared with that of a conventional Ag/AgCl (3.0 mol L^–1^ KCl) reference electrode. As shown in Figure S10 (Supplementary Data), the voltammograms recorded with both reference systems revealed a potential shift of approximately 254 mV toward more negative values when the CB/PLA pseudo-reference electrode was employed. This behavior is consistent with previous reports for 3D-printed CB/PLA-based pseudo-reference electrodes.^27,41^ Despite this potential shift, the voltammetric profiles for both references remained essentially unchanged, exhibiting comparable peak current intensities (2.01 and 2.06 μA for the CB/PLA pseudo-reference and Ag/AgCl reference electrode, respectively).

The inter-electrode reproducibility was investigated by measuring the current responses of five independent CB/PLA sensors in a 51.1 μmol L^–1^ SFT solution. From the result shown in Figure S11A (Supplementary Data), the *RSD* calculated for peak currents was 6.8%. Furthermore, the intra-electrode repeatability was evaluated through 10 consecutive measurements on a single sensor under the same conditions of reproducibility. The voltammograms presented in Figure S11B (Supplementary Data) provided an *RSD* of 2.4% for the peak currents. The results obtained suggest satisfactory reproducibility and repeatability in the analytical response, indicating good reliability for the 3D-printed platform. Additionally, the peak potential also remained highly stable throughout these experiments (*RSD* ≤ 0.5%), confirming satisfactory repeatability and reproducibility of the CB/PLA pseudo-reference electrode.

Lastly, the reproducibility of the fabrication process was evaluated using five independently fabricated sensors from different printing batches (printed in different days). As shown in Figure S12 (Supplementary Data), the peak current responses exhibited an *RSD* of 2.6%, indicating acceptable batch-to-batch reproducibility and high consistency of the additive manufacturing process.

## 4 Conclusions

This study reports, for the first time, the electrochemical determination of SFT using a portable fully 3D-printed sensing platform. A SWV-based method was successfully developed, providing rapid, sensitive, and low-cost detection of SFT in both environmental and dermal matrices. Notably, this is the first work to investigate dermal contamination as analytical focus, aiming to support the monitoring of occupational exposure in agricultural environments. Compared with previously reported electrochemical methods for SFT determination, the proposed platform achieved one of the lowest detection limits, with analytical performance comparable to that of modified sensors reported in the literature. These results are particularly relevant considering the advantages of the integrated 3D-printed electrochemical device, including portability, low fabrication cost, ease of operation, automated production and suitability for on-site analysis.

Recovery assays performed in water samples, even in the presence of some commonly co-applied agrochemicals, demonstrated satisfactory accuracy, while experiments using porcine skin confirmed the applicability of the platform for dermal exposure assessment, highlighting its potential for occupational safety applications. Overall, the proposed approach represents a practical alternative to conventional laboratory-based methods for environmental monitoring, formulation analysis, and occupational exposure assessment, contributing to the advancement of decentralized and accessible electrochemical sensing technologies for real-world applications.

## Supplementary Material



## Data Availability

The data underlying this study are not publicly available due to the confidentiality of ongoing projects. The data are available from the corresponding author upon reasonable request.

## Notes

Additional experimental results, data tables, proposed reaction mechanisms, and photographs of on-site analyses (PDF)
