# Supplementary material for: On-Site Electrochemical Detection of Sulfentrazone Using a Fully 3D-Printed Portable Sensing Platform
Source: ACS Omega. 2026 Sep 15;11(38):57259–68. doi: 10.1021/acsomega.6c05449 (PMC13628626; doi:10.1021/acsomega.6c05449)
Supplement: Supplementary file 1 [file ao6c05449_si_001.pdf]

Supplementary Data for

On-site electrochemical detection of sulfentrazone using a fully 3D-printed portable sensing platform

Jacqueline Marques Petroni [a, b, *], Jonas Raul Balbinoti [b], Elias Jorge [c], Osvaldo Cavalcante da Costa Neto [c], Rodrigo Amorim Bezerra da Silva [d], Magno Aparecido Gonçalves Trindade [e, f] and Bruno Gabriel Lucca [a, *]

[a] Institute of Chemistry, Federal University of Mato Grosso do Sul, Campo Grande, MS, 79074-460, Brazil.

[b] Occupational Health and Safety Management Systems, Industry Social Service Innovation Center, Campo Grande, MS, 79002-072, Brazil.

[c] Occupational Health Department, Eldorado Brasil, Três Lagoas, MS, 79641-300, Brazil.

[d] Institute of Chemistry, Federal University of Uberlandia, Uberlandia, MG, 38400-902, Brazil.

[e] Faculty of Exact Sciences and Technology, Federal University of Grande Dourados, Dourados, MS, 79804-970, Brazil.

[f] Department of Fundamental Chemistry, Institute of Chemistry, Universidade de São Paulo, São Paulo, SP, 05508-900, Brazil.

Corresponding Authors:

* Dr. Jacqueline Marques Petroni
Institute of Chemistry, Federal University of Mato Grosso do Sul, Campo Grande, MS, Brazil, 79074-460
Phone: +55 (67) 3345-3936
Email: jacque_petroni@hotmail.com

* Prof. Dr. Bruno Gabriel Lucca
Institute of Chemistry, Federal University of Mato Grosso do Sul, Campo Grande, MS, Brazil, 79074-460
Phone: +55 (67) 3345-3936
Email: bruno.lucca@ufms.br

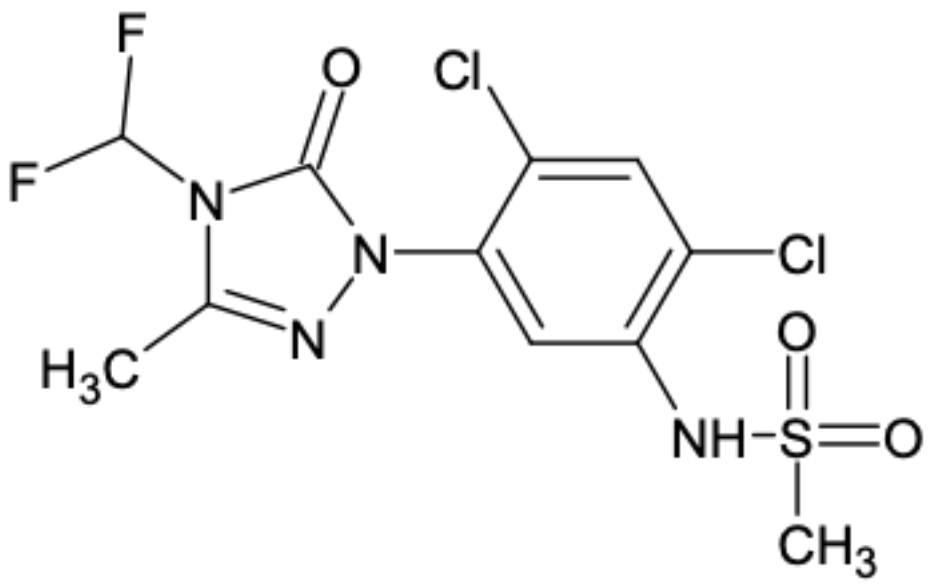

Fig. S1. Sulfentrazone chemical structure.

Table S1. Active ingredients of the commercial products used as interferents in the SFT analysis, along with the preparation conditions employed for eucalyptus cultivation and interference studies. The dilution ratio and pH of each commercial product are provided to facilitate interpretation of the results.

Active Principle	Recommended dissolution [1]	[Recommended][2]	[Used]	Ratio [3] [SFT]:[AP]	pH [4]
**SFT**	**0.6 L**	**3 g L^{-1}**	**0.03 g L^{-1}**	–	–
ACT	60 g	0.15 g L^{-1}	0.0015 g L^{-1}	1:0.2	5.29
APP	6 L	2.22 g L^{-1}	0.022 g L^{-1}	1:0.73	5.5 – 6.5
BIF	60 g	0.15 g L^{-1}	0.0015 g L^{-1}	1:0.2	5.29
CFN	0.10 L	0.24 g L^{-1}	0.0024 g L^{-1}	1:0.08	6.0 – 8.0
GLY	750 g	5.4 g L^{-1}	0.054 g L^{-1}	1:1.8	4.22
IDZ	0.75 L	0.375 g L^{-1}	0.0037 g L^{-1}	1:0.12	7.0 – 8.5
ISX	50 g	0.375 g L^{-1}	0.0037 g L^{-1}	1:0.12	4.0 – 6.0
PHC	1 L	0.92 g L^{-1}	0.0092 g L^{-1}	1:0.31	Not applicable
PYR	0.10 L	0.25 g L^{-1}	0.0025 g L^{-1}	1:0.08	5.0 – 7.0
SFR	6 g	0.012 g L^{-1}	0.00012 g L^{-1}	1:0.004	4.9
TMX	150 g	0.375 g L^{-1}	0.0037 g L^{-1}	1:0.25	7.0 – 11.0

[1] Recommended dissolution according to the leaflet of the commercial product for eucalyptus cultivation from 100 L of H_2O.
[2] Concentration of active ingredient in the pesticide syrup from the recommended dissolution, considering the active ingredient content in the commercial product.
[3] Ratio of [Sulfentrazone]: [Active Principle].
[4] pH of commercial product at 20 ºC.

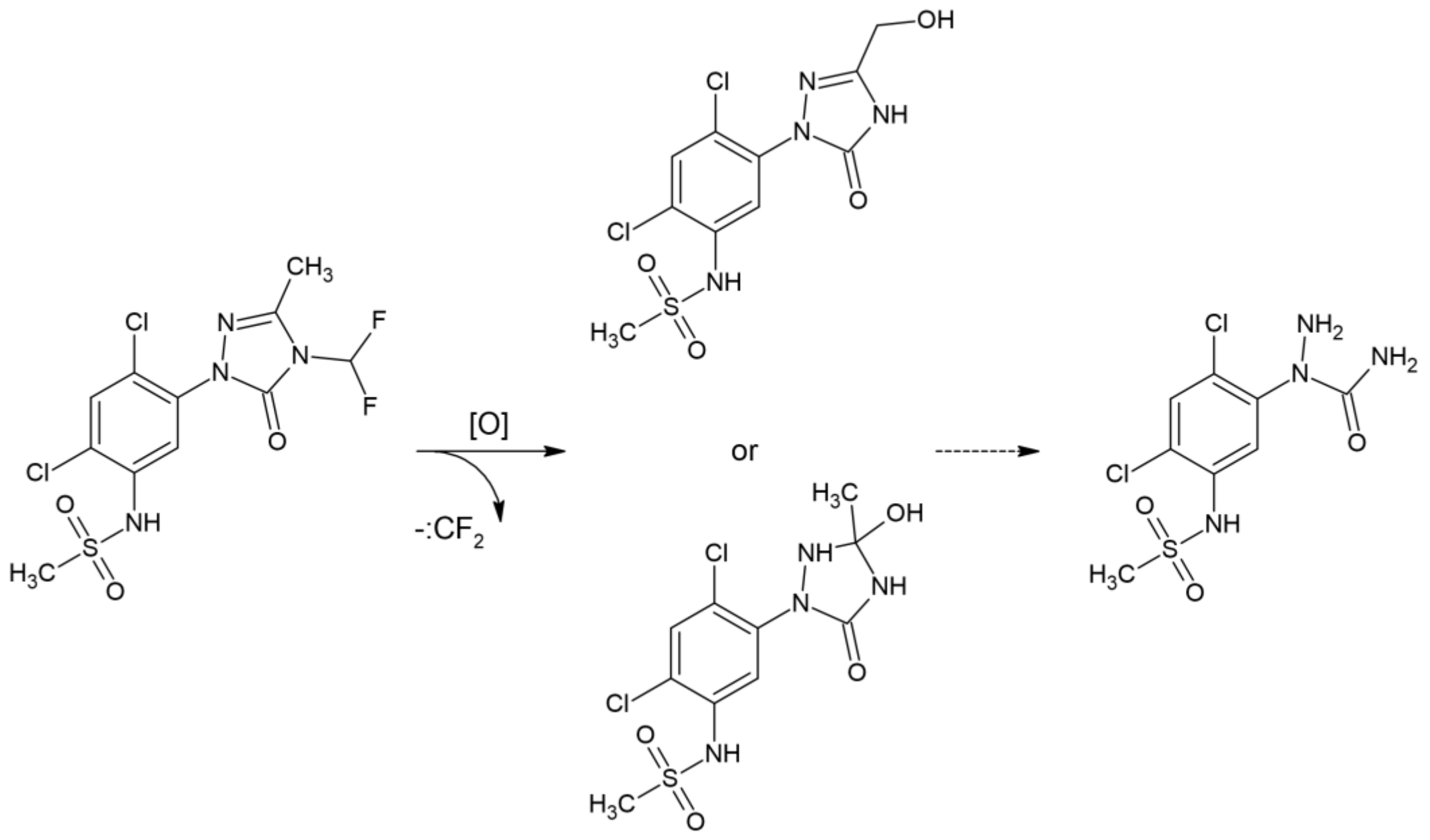

Fig. S2. A possible SFT oxidation mechanism, as proposed by Lima et al. (https://doi.org/10.1016/j.chemosphere.2010.08.003).

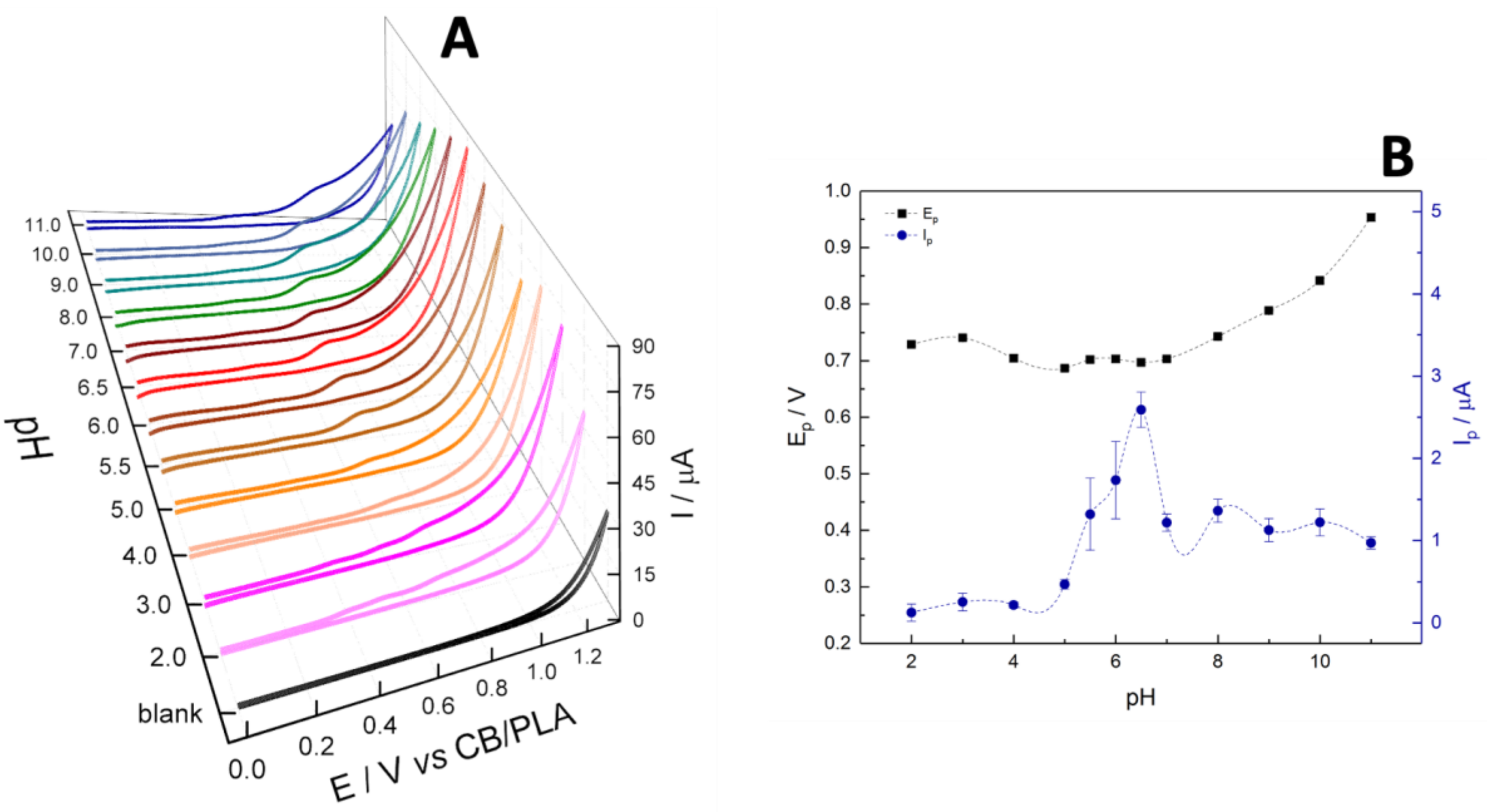

Fig. S3. (A) Cyclic voltammograms in 0.040 mol L^{-1} BR buffer in absence and presence of 210 µmol L^{-1} SFT for different pH values (2.0 to 11.0) recorded on CB/PLA and (B) respective plot of peak currents and peak potentials as function of pH (mean ± standard deviation, results for n = 3 measurements). Experimental conditions: ΔE_s = 2 mV, ν: 100 mV s^{-1}.

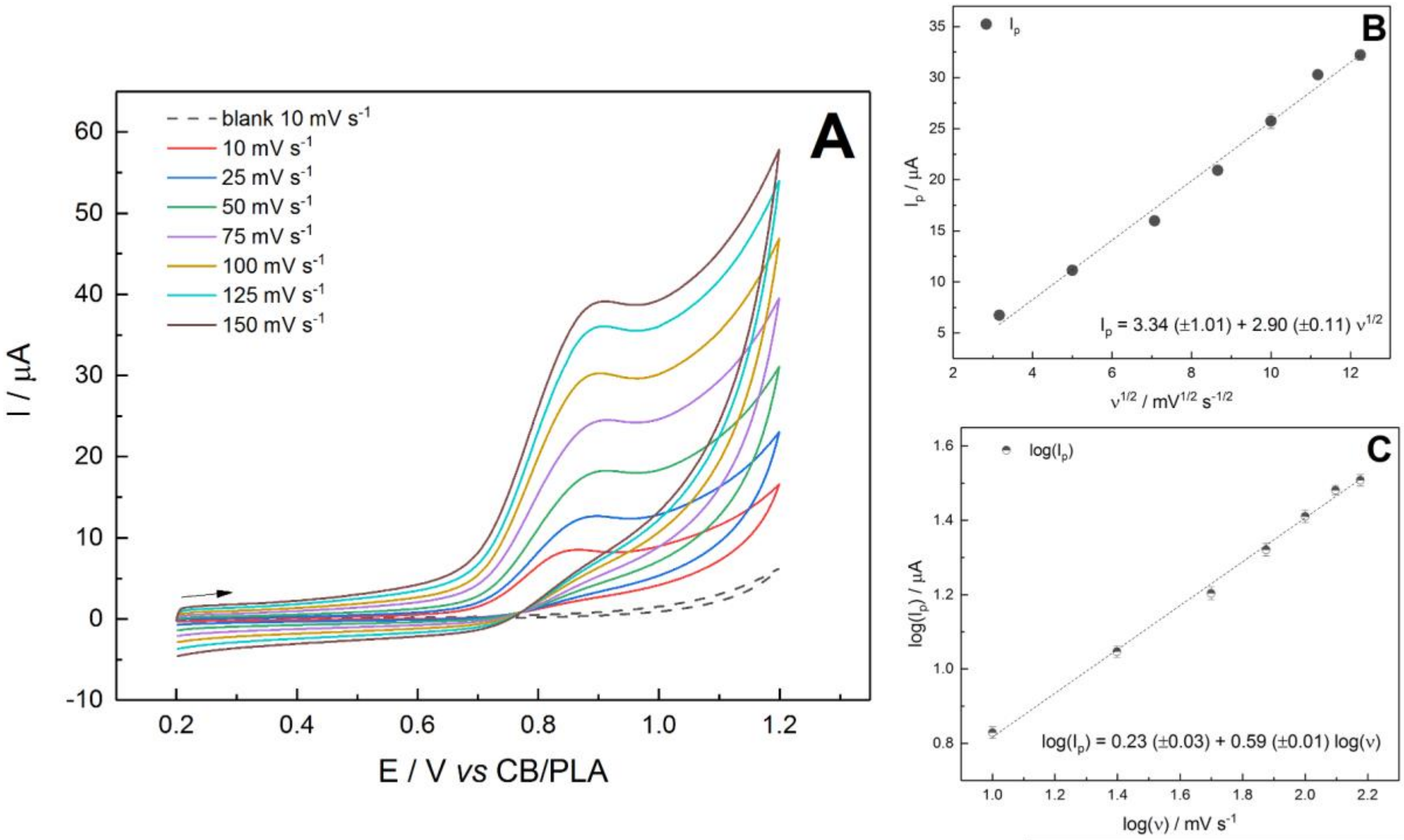

Fig. S4. (A) Cyclic voltammograms in 0.040 mol L^{-1} BR buffer (pH 6.5) in absence and presence of 653 µmol L^{-1} SFT at different scan rates (10 to 150 mV s^{-1}). Plots of (B) peak currents as function of the square root of scan rates and (C) dependence of the logarithm of the peak currents on logarithm of the scan rate. Mean ± standard deviation, results for n = 3 measurements.

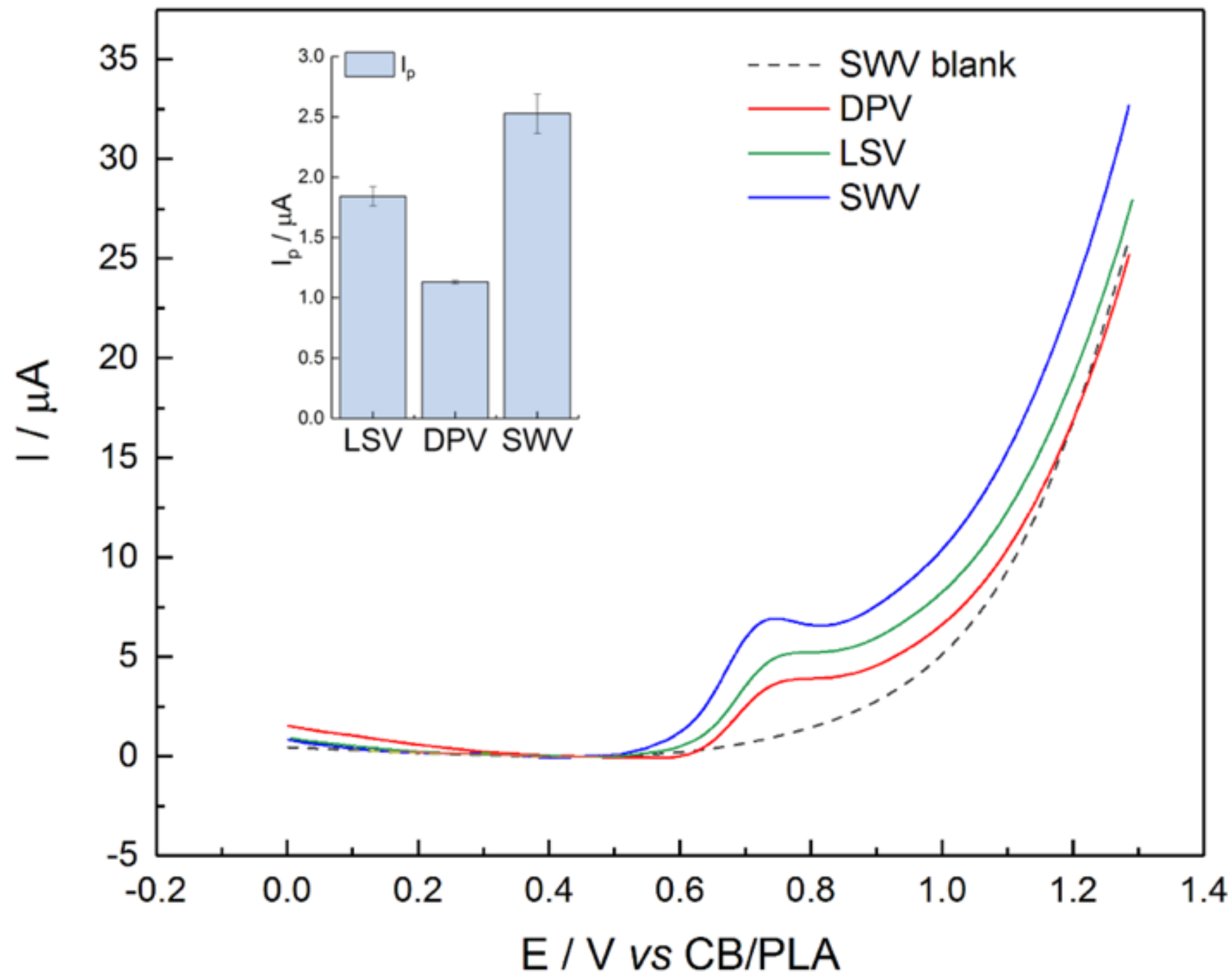

Fig. S5. Voltammograms recorded in the absence (dashed line, just for SWV) and the presence of 210 µmol L^{-1} of SFT using DPV (red line), LSV (green line) and SWV (blue line). Inserted graphic: Comparison of the peak currents extracted from the voltammograms (mean ± standard deviation, results for n = 3 measurements). Parameters: electrolyte was 0.04 mol L^{-1} BR buffer (pH = 6.5); ΔE_S = 5 mV; a = 20 mV; ν = 50 mV s^{-1}.

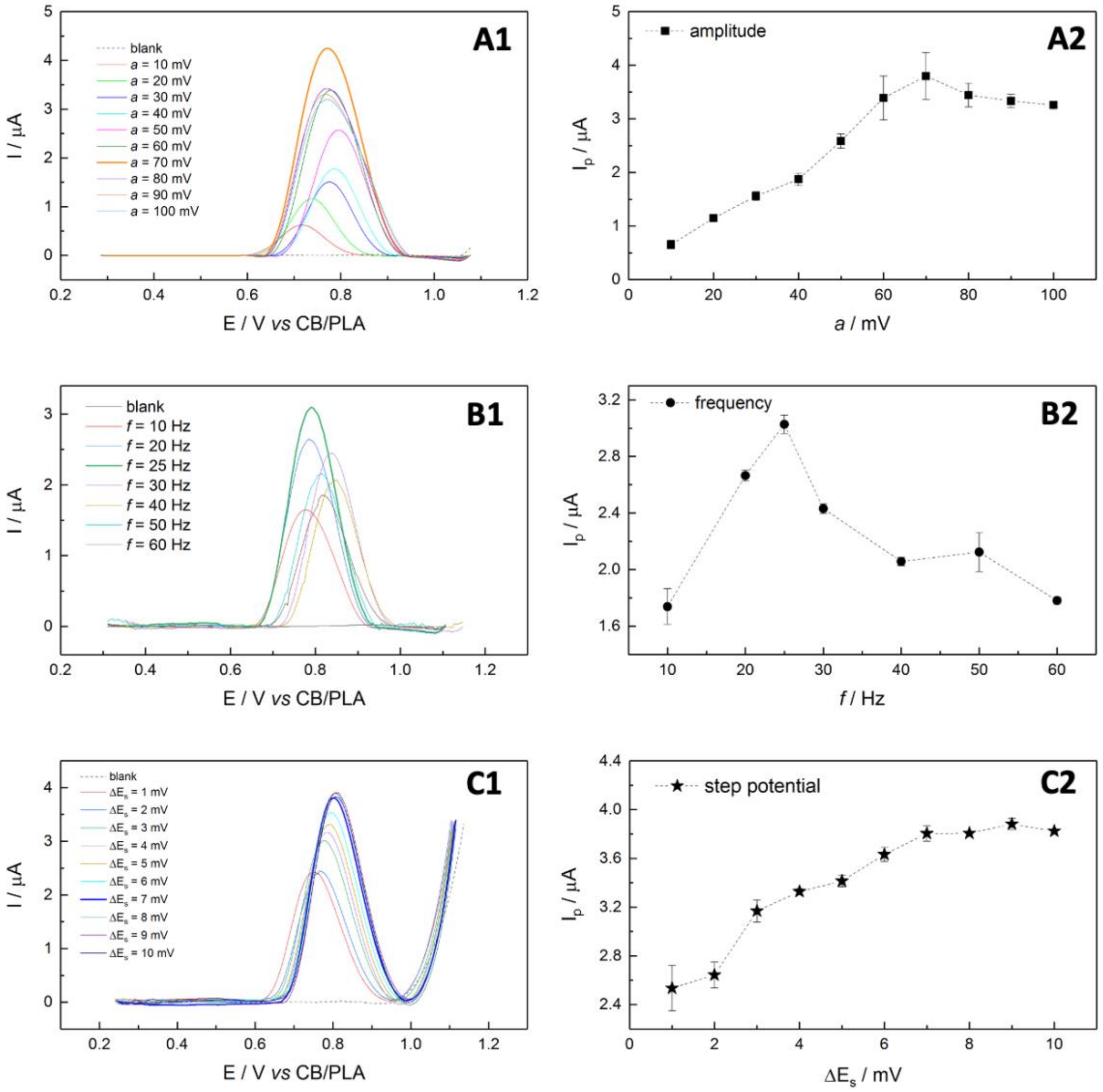

Fig. S6. Optimization of SWV parameters using 210 µmol L^{-1} of SFT in 0.04 mol L^{-1} BR buffer pH 6.5: (A1) voltammograms and (A2) plot of SFT peak currents as a function of amplitude (*a*). Parameters used: $f = 10$ Hz and $\Delta E_s = 5$ mV; (B1) voltammograms and (B2) plot of SFT peak currents as a function of frequency (*f*). Parameters used: $a = 70$ mV (optimized value) and $\Delta E_s = 5$ mV; and (C1) voltammograms and (C2) plot of SFT peak currents as a function of step potential (ΔE_s). Parameters used: $a = 70$ mV and $f = 25$ Hz (optimized values). Results for $n = 3$ measurements (mean ± standard deviation).

Table S2. Parameters optimized for the electroanalytical detection of SFT using SWV and CB/PLA 3D-printed sensor.

Parameters	Range studied	Value optimized
**Amplitude (*a*)**	10 - 100 mV	70 mV
**Frequency (*f*)**	10 - 60 Hz	25 Hz
**Step potential (ΔE_s)**	1 - 10 mV	7 mV

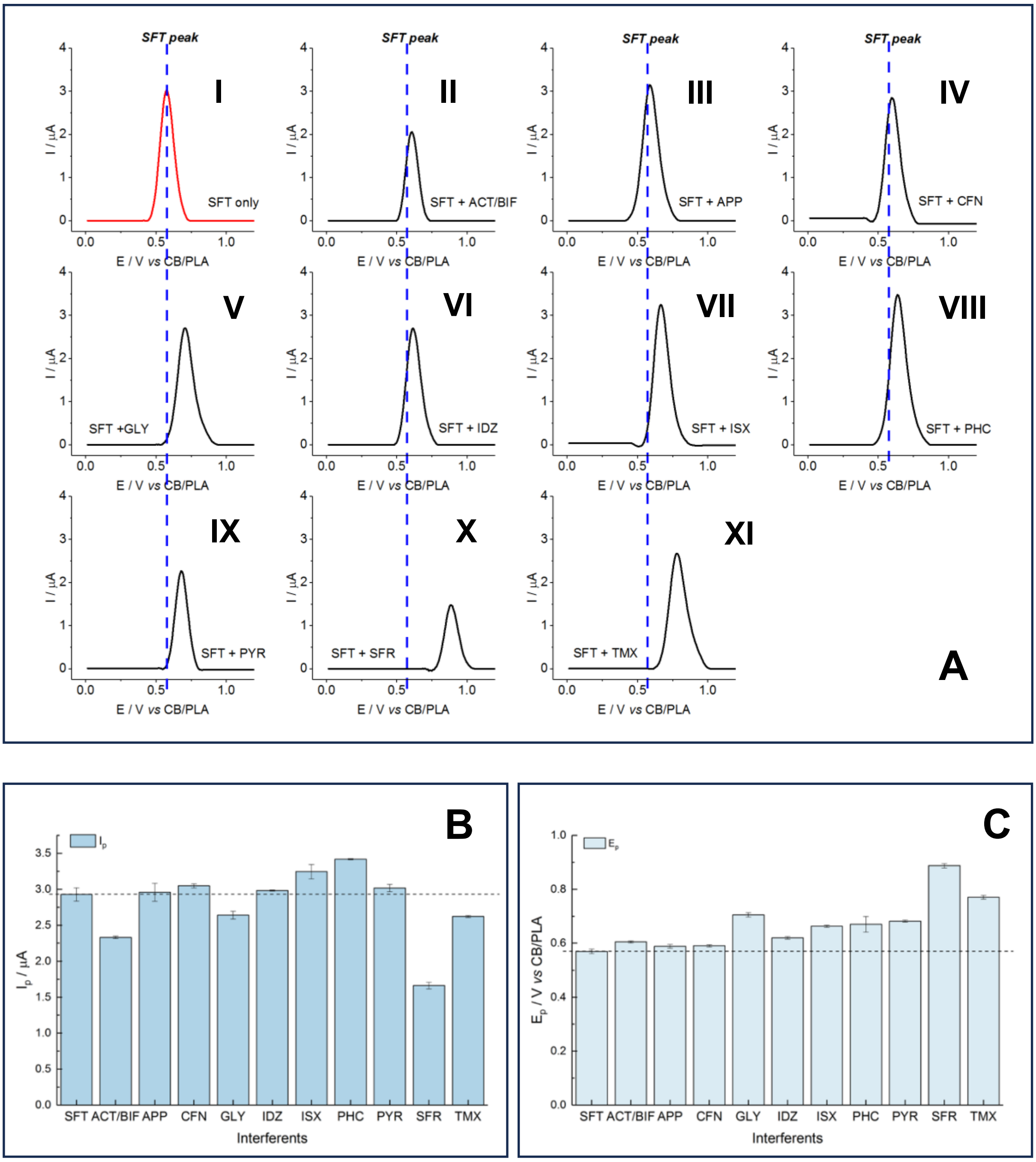

Fig. S7. A) SW voltammograms obtained for the study of interferents from commercial agrochemical formulations: I) sulfentrazone only (control); II): sulfentrazone + acetamiprid + bifenthrin; (III) sulfentrazone + ammonium polyphosphate; (IV) sulfentrazone + chlorfenapyr; (V) sulfentrazone + glyphosate; (VI) sulfentrazone + indaziflam; (VII) sulfentrazone + isoxaflutole; (VIII) sulfentrazone + paraffinic hydrocarbons; (IX) sulfentrazone + pyraclostrobin; (X) sulfentrazone + sulfluramid; and (XI) sulfentrazone + thiamethoxam. Graphs showing the variation of B) peak current and C) peak potential for each interferent studied. Experimental conditions as in Table S2 (Supplementary Data).

Table S3. Agrochemicals included in the controlled mixtures used for the recovery assays and their concentration ratios relative to sulfentrazone (SFT).

Active Ingredient	Mass Ratio [SFT]:[Active Ingredient] [1]
**SFT**	–
GLY	1:1.84
APP	1:0.75
ISX	1:0.09
PHC	1:0.21
TMX	1:0.25

[1] The concentration ratios were defined according to the application rates recommended by the manufacturers for eucalyptus cultivation.

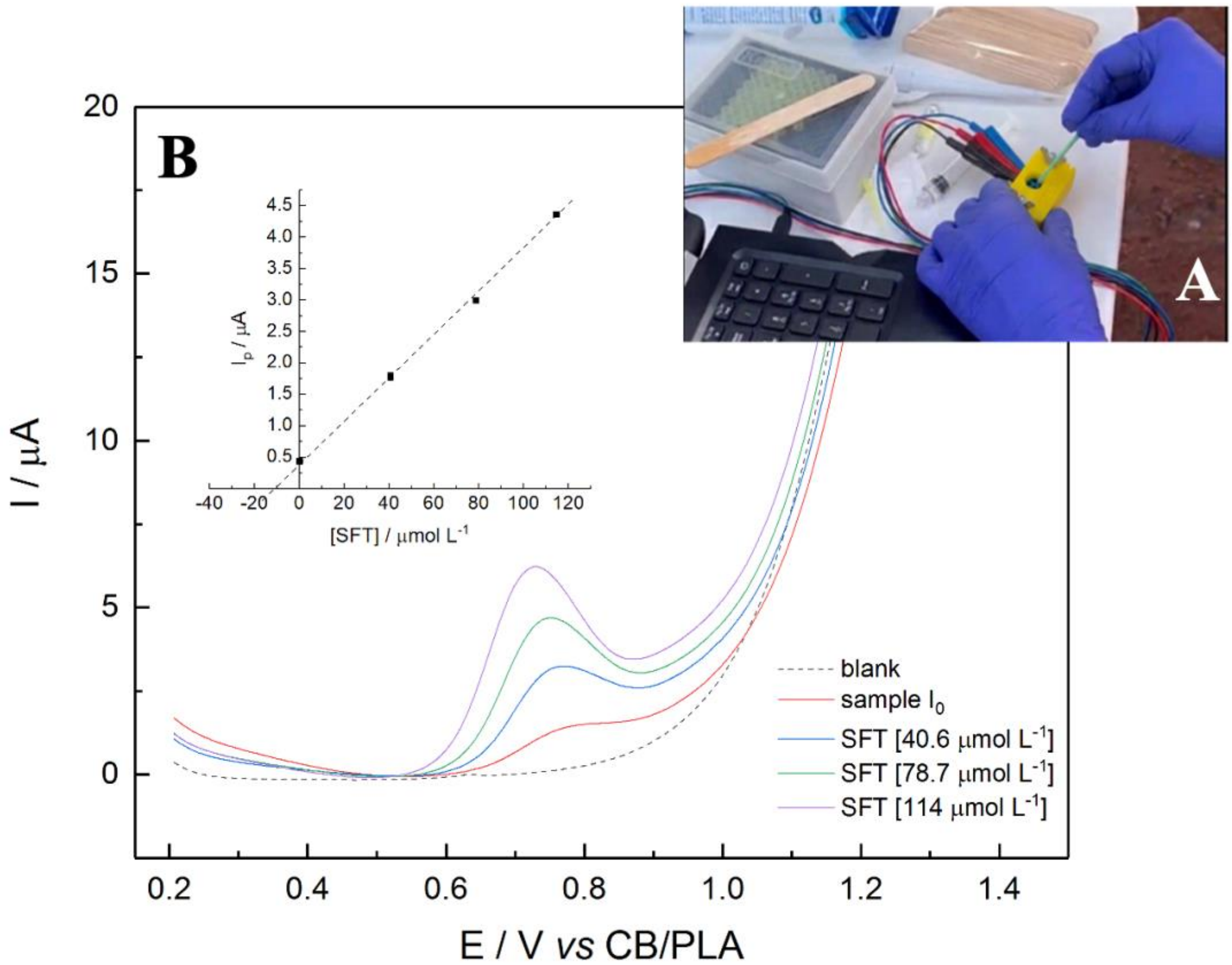

Fig. S8. (A) Portable electrochemical platform used during the on-site measurements. (B) Representative SW voltammogram obtained for the recovery assay of SFT in the presence of commonly co-applied agrochemicals (GLY, APP, ISX, PHC and TMX). The inset shows the corresponding standard addition plot (mean ± SD, n = 3). Experimental conditions are described in Table S2.

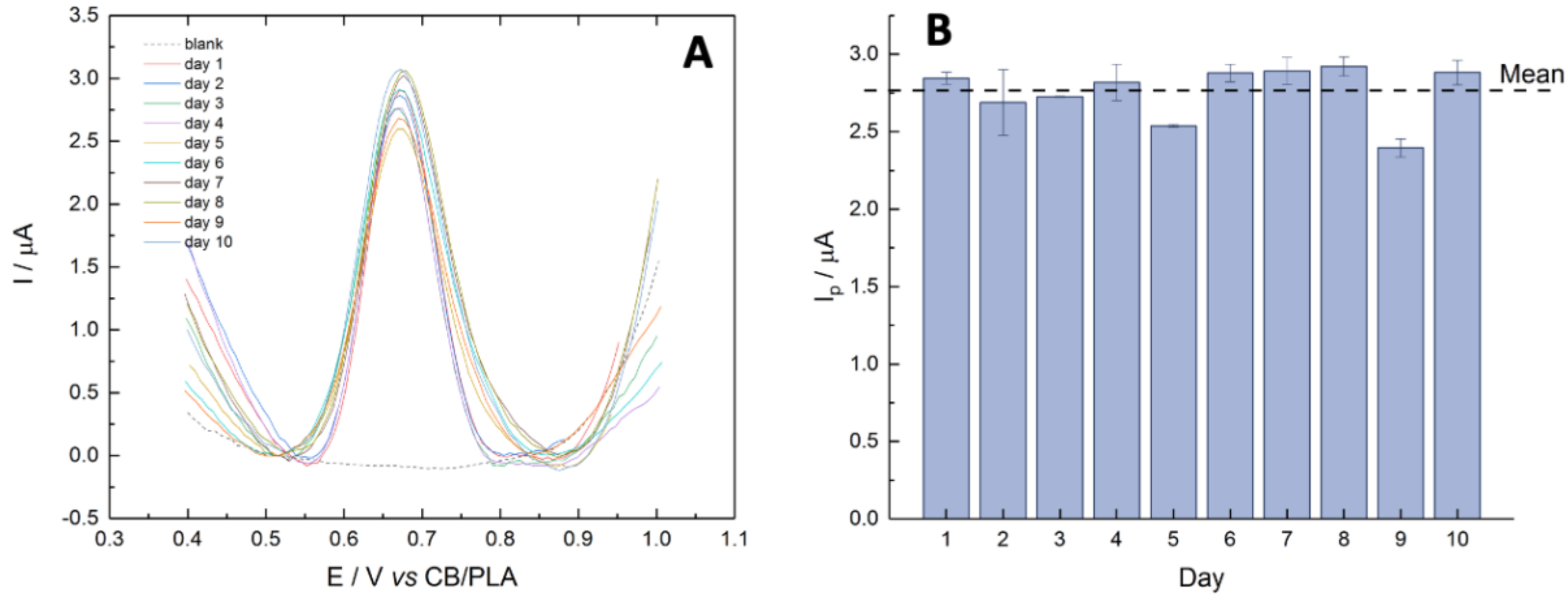

Fig. S9. (A) SW voltammograms of 53.8 µmol L^{-1} SFT recorded during a period of 10 days using the same solution and the same electrode. (B) Graph showing the variation of peak currents over the days. Experimental conditions as in Table S2 (Supplementary Data).

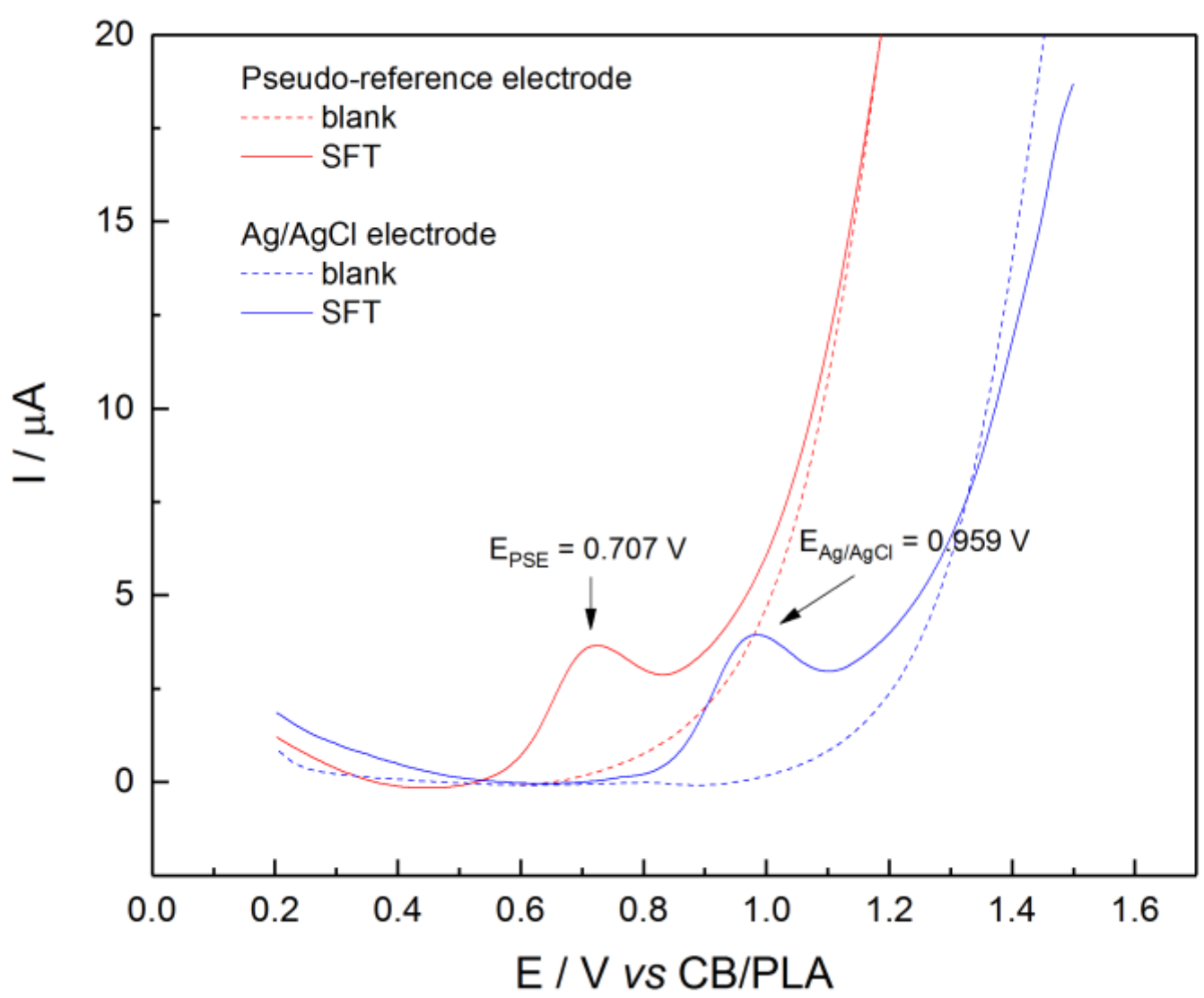

Fig. S10. SW voltammograms of 30.7 µmol L^{-1} SFT recorded with the CB/PLA pseudo-reference electrode (red lines) and Ag/AgCl reference electrode (blue lines). The working and counter electrodes were the same for all measurements.

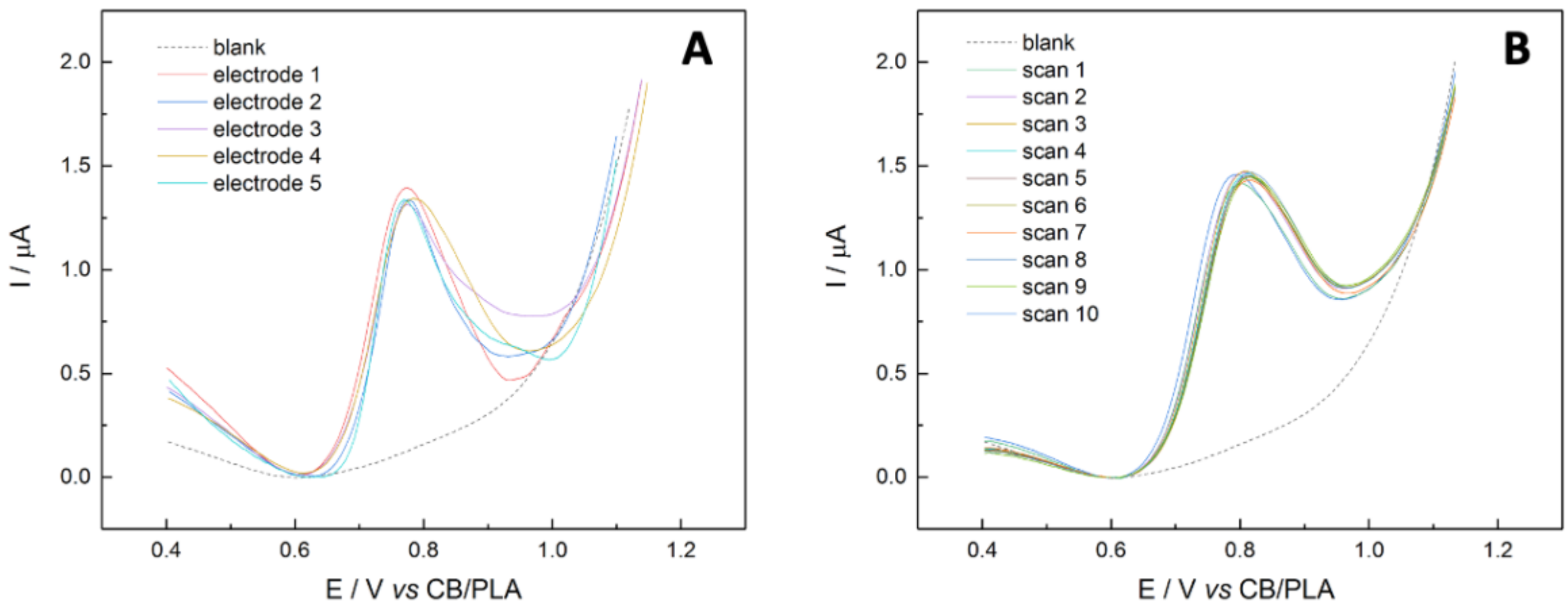

Fig. S11. SW voltammograms of 51.1 µmol L^{-1} SFT using A) five different CB/PLA electrodes and B) a single electrode for successive measurements (n = 10). Experimental conditions as in Table S2 (Supplementary Data).

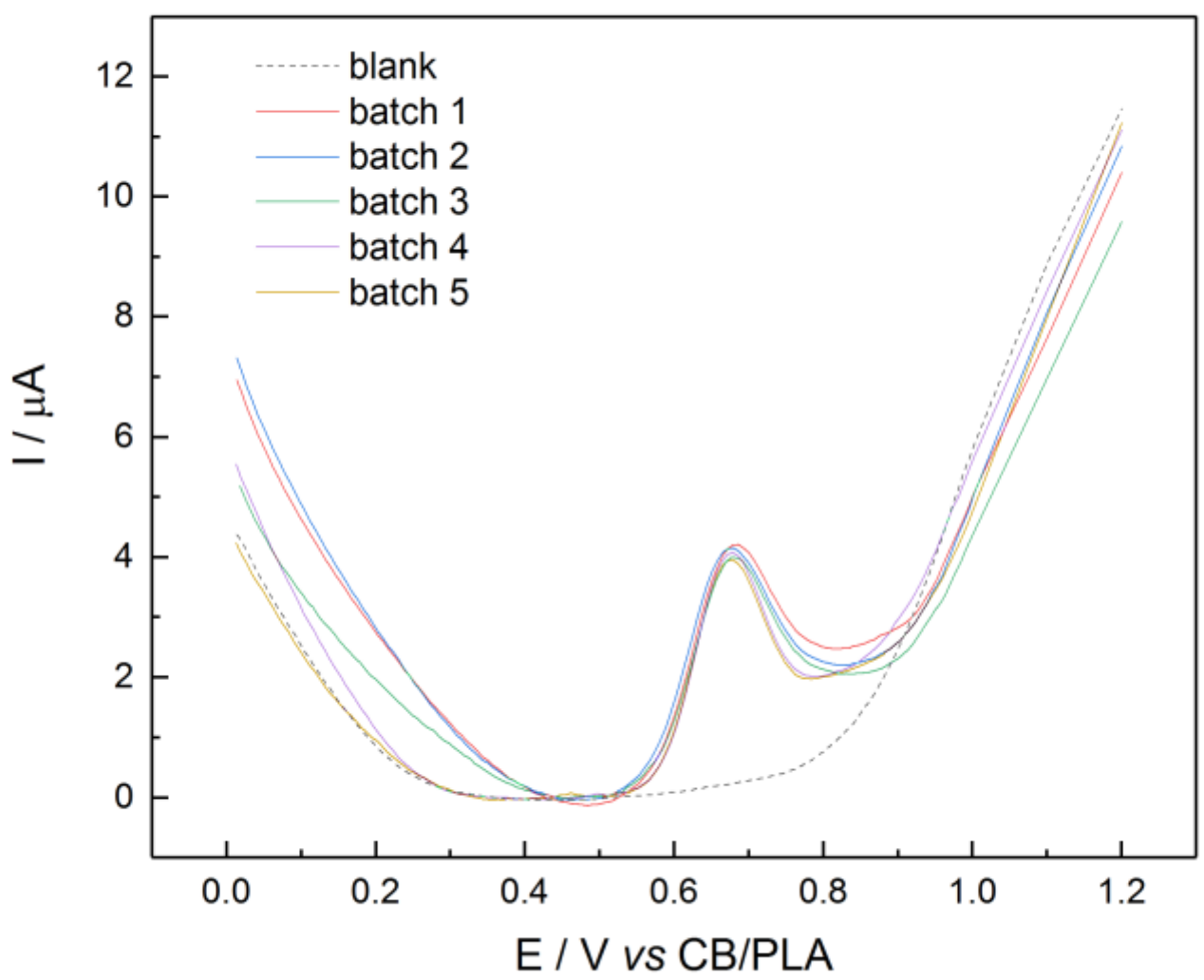

Fig. S12. SW voltammograms of 51.1 µmol L^{-1} SFT recorded using sensors from five different batches. Experimental conditions as in Table S2 (Supplementary Data).
